# One‐Step Synthesis of Chitosan Hydrogel as Electrochemical Chemosensor for Hydrogen Sulfide Detection in Pregnancy‐Induced Hypertension Syndrome Serum Sample

**DOI:** 10.1002/open.202400107

**Published:** 2024-06-04

**Authors:** Lishan Fang, Jinqiu Li, Wei Lin, Lili Zeng, Liumin Yu, Zhanfei Chen, Jianlin Shen, Yu Chen, Zhonghui Chen, Zhenyu Lin

**Affiliations:** ^1^ Department of obstetrics Affiliated Hospital of Putian University Putian University Putian 351100 China; ^2^ Central laboratory Affiliated Hospital of Putian University Putian University Putian 351100 China; ^3^ Ministry of Education Key Laboratory for Analytical Science of Food Safety and Biology Department of Chemistry Fuzhou University Fuzhou 350116 China; ^4^ Department of rehabilitation medicine Affiliated Hospital of Putian University Putian University Putian 351100 China

**Keywords:** Hydrogen sulfide detection, Hydrogel, Copper ion, stimulus-response, Pregnancy-induced hypertension syndrome

## Abstract

Oxidative stress caused by pregnancy‐induced hypertension syndrome significantly affects the health of pregnant women. Hydrogen sulfide is a typical gaseous signal molecule against oxidative stress, and it is of profound significance to develop a detection method. In this study, a stimuli‐responsive hydrogel was constructed based on the coordination and bonding principle of metal ions and chitosan (CS) to realize the quantitative detection of hydrogen sulfide (H_2_S). The chain of CS contains a large number of amino groups and hydroxyl groups, which can form the coordination structure with Cu^2+^, triggering CS to form a stable hydrogel. The hydrogel can be formed within about 5 s, which has the characteristics of rapid preparation. In the presence of target H_2_S, the cross‐linking agent Cu^2+^ in the hydrogel can compete out, resulting in the collapse of the hydrogel and the release of the electrochemical probe. By detecting the concentration of the released electrochemical probe, the quantitative detection of H_2_S can be achieved. The prepared hydrogel has a good linear relationship with the concentration of H_2_S from 1 μM to 60 μm. At the same time, the hydrogel has good specificity and stability, and it can be applied to the detection of H_2_S in serum samples.

## Graphical abstract

A hydrogen sulfide (H_2_S) stimuli‐responsive hydrogel was constructed based on the coordination and bonding principle of metal ions and chitosan (CS). The hydrogel can be rapidly prepared within 5 s. In the presence of H_2_S, the cross‐linking agent Cu^2+^ can compete out, and release of the electrochemical probe. The prepared hydrogel has a good linear relationship with H_2_S.

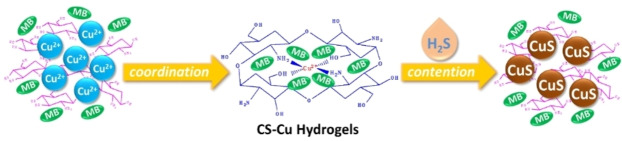

## Introduction

Pregnancy‐induced hypertension syndrome (PIH) is a particular disease of pregnant women with complex etiology and serious harm. The incidence of the PIH disease in pregnant women is about 5–10 %, and it is one of the important risk factors leading to maternal and neonatal mortality.^[1]^ It has been observed that gaseous signal molecules are the typical endogenous gas molecules with important physiological functions, including carbon monoxide (CO), nitric oxide (NO), and hydrogen sulfide (H_2_S).^[2]^ This kind of molecule has the characteristics of small molecular weight, continuous production, rapid diffusion, and extensive action. It can significantly regulate vascular relaxation and is closely related to the pathogenesis of PIH. Hydrogen sulfide is a representative gaseous signal molecule. It has been proven to play a positive role in regulated vascular dilatation, and corrected the imbalance of proliferation and apoptosis of vascular smooth muscle cells in spontaneous hypertension and pulmonary hypertension.^[3]^ Therefore, the determination of hydrogen sulfide concentration in PIH patients is of great clinical significance for the detection, diagnosis, and treatment of gestational hypertension and the safety of pregnant women and their fetuses.

To understand the role of H_2_S in various diseases pathophysiology, several detection methods have been created, including electrochemical detection,^[4]^ colorimetric analysis,^[5]^ fluorescence probe,^[6]^ and surface‐enhanced Raman scattering.^[7]^ Compared with other H_2_S detection methods, the electrochemical detection method has unique‐particular advantages of easy synthesis and quick response.^[8]^ Nowadays, the newly emerged electrochemical detection method, especially the ‘signal‐on’ strategy, has attracted much focus for H_2_S detection. Zhang et al. successfully created an integrated online electrochemical system based on microchips for H_2_S detection.^[9]^ By this method, Ru(NH_3_)_6_^3+^ and H_2_S have been coupled with electrochemical chemical reactions and shown well properties. Huang et al. developed an amperometric method for H_2_S detection through 3D specialized material.^[10]^ Based on the structure of the electrode material, they monitored long‐time and real‐time H_2_S released from HeLa cells stimulated with cysteine. Therefore, selective detection of H_2_S can be achieved by constructing an electrochemical‐specific recognition interface.

In this study, a H_2_S‐stimulated hydrogel has been constructed, and the detection signal was implemented by signal transduction. Chitosan (CS) has a strong affinity for metal ions, which can form a coordination structure with CS and exist in the form of [Mn^+^(NH_2_)_n_]^n+^ under weakly acidic conditions.^[11]^ It exists as [Mn^+^(NH_2_)]^n+^ in the neutral environment. Moreover, it shows different coordination abilities to different metal ions, that is Cu^2+^≫Hg^2+^>Zn^2+^>Ni^2+^>Co^2+^>Ca^2+^. CS and metal ions form a polymer hydrogel in a certain environment, and the hydrogel can retain a large amount of molecules without structure dissociation. This type of hydrogel has good hydrophilicity and biocompatibility, and it can produce stimulus responses to the external environment or target.^[12]^ Based on the above principles, Cu^2+^ has served as a cross‐linking agent for the hydrogel to trigger the polymerization of CS to form the Cu−CS hydrogel, and an electrochemical probe methylene blue (MB) was embedded in the Cu−CS hydrogel structure. In the absence of target H_2_S, Cu−CS could exist stably in solution without dissociation and could not release MB. In the presence of target H_2_S, the cross‐linking agent Cu^2+^ in the hydrogel can be competed out of hydrogel, and released MB. By detecting the concentration of MB, H_2_S can be quantitatively detected.

## Experimental Section

### Materials and Chemicals

Sodium sulfide nonahydrate (Na_2_S ⋅ 9H_2_O), chitosan (CS), cupric sulfate (CuSO_4_), acetic acid glacial (CH_3_COOH), sodium hydroxide (NaOH), methylene blue (MB), kalium bromatum (KBr), sodium chloride (NaCl) and kalium chloratum (KCl) were purchased from Aladdin (Shanghai, China). All reagents were of analytical‐regent (A. R.) grade and directly used for the following experiments without further purification. Ultrapure water obtained from the Millipore water purification system (18.2 MΩ⋅cm, Mill‐pore) was used in all experiments.

### Instruments

The electrochemical measurements were recorded by an electrochemical workstation (CHI660D, Chenhua Instruments, Shanghai, China) with a classical three‐electrode system. Then, a glassy carbon electrode (GCE, diameter: 3 mm, 99.99 % (w/w) polycrystalline, Chenhua Instruments, Shanghai, China) was employed as the working electrode, Ag/AgCl electrode saturated with 3 M KCl served as the reference electrode, and platinum wire used as the counter electrode. Field emission scanning electron microscope (SEM, S‐4800, Hitachi, Japan) was used to study the morphology of the CS hydrogel. Infrared spectroscopy was recorded with the fourier infrared spectrometer (FT‐IR, Nicolet 6700, Thermo Scientific, USA).

### Preparation of CS hydrogels

Firstly, prepare 0.5 wt% CS solution. 0.025 g of CS solid was weighed into a 10 mL centrifuge tube, and 5 mL of 0.2 % acetic acid solution was added. The CS solid was completely dissolved through a vortex mixer, and the bubbles in the solution were removed by ultrasound. Then, 20 μL of 1 mM methylene blue solution was added to the CS solution. The mixture of CS and MB (300 μL) was put into a 2 mL centrifuge tube, and CuSO_4_ was added as the triggering agent of the hydrogel. The aqueous CS solution was rapidly mixed on a vortex mixer, and the CS aqueous solution could rapidly polymerize to form Cu−CS hydrogel within 5 s. Finally, distilled water was added to remove the unembedded MB, and the water in the Cu−CS surface was absorbed by filter paper.

### Study on H2S Stimuli‐Responsive Hydrogel

According to the previous studies and literature, Na_2_S was used as the donor of H_2_S. The different concentrations of H_2_S standards were obtained by stepwise dilution. The H_2_S standard was added to the Cu−CS hydrogel, mixed through the vortex mixer, and incubated at room temperature. At this time, H_2_S and Cu−CS hydrogel have a competitive reaction, that is, Cu^2+^ in the hydrogel reacts with S^2−^ to generate CuS, which leads to the collapse of the Cu−CS hydrogel structure and the release of MB. Then, the concentration of H_2_S can be obtained by measuring the electrochemical signal of MB.

In this experiment, the concentration of MB was determined to quantitatively detect H_2_S in the samples. Quantitative detection of MB was performed by differential pulse voltammetry (DPV). Firstly, GCE (3 mm) was polished with 1.0, 0.3, and 0.05 μm Al_2_O_3_ powder, and sonicated with ethanol, piranha wash, and distilled water. Then, the treated GCE was immersed in 0.5 M H_2_SO_4_ solution, Ag/AgCl was used as the reference electrode, and platinum wire was used as the counter electrode. The cyclic voltammetry was used to scan the electrode in the potential range of −0.2 to 1.0 V until a stable cyclic voltammetry was obtained. The GCE was rinsed with distilled water and set aside. Finally, GCE was placed in the above test solution containing MB, and it was quantitatively detected by the DPV method. The detection conditions were as follows: pulse amplitude: 0.05 V; Pulse width: 0.05 s; The potential ranged from −0.05 to 0.35 V.

The serum samples used in the proposed Cu−CS hydrogel were provided by the Affiliated Hospital of Putian University, which were normal human and PIH patients’ serum samples. The experiment was conducted in strict accordance with the protocol approved by the Ethics Committee of the Affiliated Hospital of Putian University. The procedure for testing serum samples is the same as above.

## Results and Discussion

### Principle of the Proposed H_2_S Stimuli‐Responsive Hydrogel

**Scheme 1:**
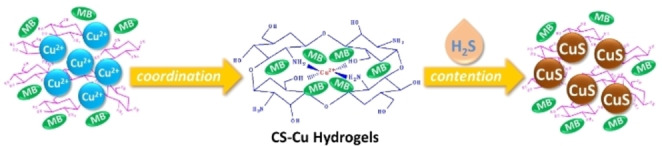
Schematic illustration for H_2_S detection based on one‐step formation of chitosan hydrogel.

Scheme 1 shows the experimental schematic illustration of Cu−CS hydrogel rapidly synthesized in one step for H_2_S detection. Firstly, the CS solution was mixed evenly with the electrochemical probe MB. Since chitosan contains a large number of amino groups and hydroxyl groups in the molecular chain, Cu^2+^ can coordinate with CS to form hydrogels. Then, Cu^2+^ was used as the triggering agent to form a Cu−CS hydrogel polymer, and MB was embedded in the 3D space of the hydrogel. In the presence of the target Na_2_S, it can complexate with Cu^2+^ in Cu−CS hydrogel to form CuS precipitates.

In the presence of the target, Na_2_S can compete with Cu^2+^ in Cu−Cs hydrogel to form the precipitate CuS. This substance has a low precipitation solubility product constant (K_sp_ CuS=6.3×10^−36^), which leads to Cu^2+^ competing out of the Cu−CS hydrogel, resulting the three‐dimensional structure of the hydrogel destruction and release of MB. Finally, the quantitative analysis and detection of H_2_S can be realized by detecting the electrochemical signal of MB by DPV, and this method is a signal‐enhanced detection method.

### Characterization of CS Hydrogel

**Figure 1:**
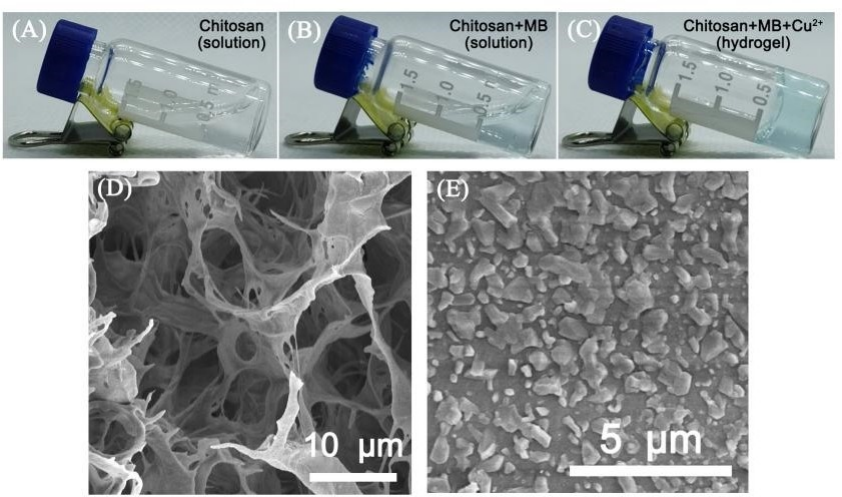
The hydrogel transformation from CS solution to Cu−CS hydrogel, (A) Chitosan, (B) Chitosan+MB, (C) Chitosan+MB+Cu^2+^; Photograph of the prepared hydrogel SEM images in (D) absence and (E) presence of H_2_S of Cu−CS hydrogel.

According to the photograph of the hydrogel preparation process, the chitosan aqueous solution appears as a liquid state (Figure 1A). The aqueous chitosan solution turned light blue when 0.5 μg/mL MB molecules were added (Figure 1B). Then, Cu^2+^ was added to the above solution, chitosan was sent to polymerize with Cu^2+^ to form a solid hydrogel (Figure 1C). Then, SEM was used to characterize the morphological changes of Cu−CS hydrogel in H_2_S stimuli‐responsive. As shown in Figure 1D, the morphology of the fabricated hydrogel was investigated by SEM, and the supramolecular hydrogel shows typical porous structure, indicating the hydrogel was successfully synthesized. When 60 μM H_2_S was added, the SEM image can be shown that a large amount of solid was generated on the hydrogel surface (Figure 1E). The experimental results show that, since Cu^2+^ in hydrogel reacts with H_2_S to form CuS after adding H_2_S, the Cu−Cs hydrogel rapidly disintegrates.

**Figure 2:**
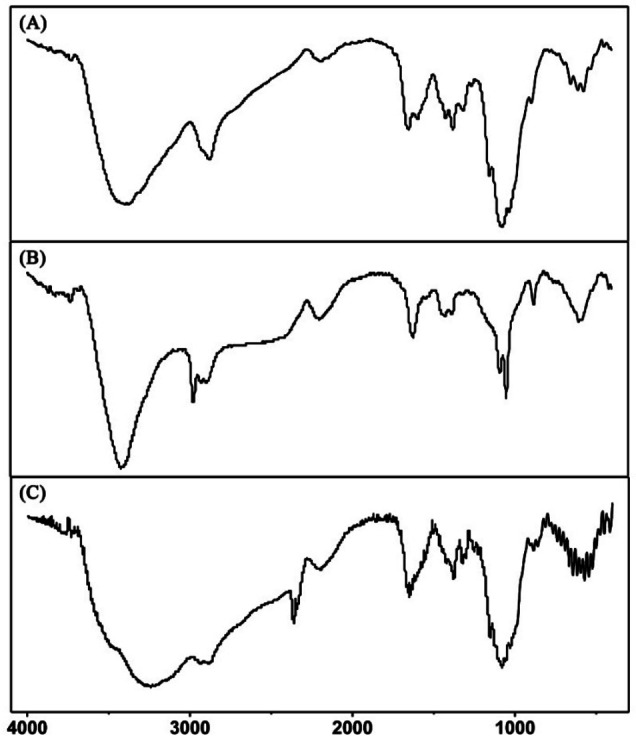
FT‐IR spectra of (A) CS powders, (B) Cu−CS hydrogel, and (C) Cu−CS hydrogel stimulated with H_2_S.

To further explore the changes of Cu−CS hydrogel before and after H_2_S stimulation response, FT‐IR was used to characterize it. First, the synthesized Cu−CS hydrogel was flash‐frozen in liquid nitrogen and transferred to a vacuum freeze dryer until completely lyophilized. Then, the solid hydrogel and KBr were ground and mixed evenly, and pressed into the machine for testing. The FT‐IR spectra of the CS powder are shown in Figure 2A, the absorption peaks of ν_N‐H+O‐H_ at 3400 cm^−1^, δ_N‐H_ at 1590 cm^−1^, and ν_C‐OH_ at 1080 cm^−1^. After the amino group and hydroxyl group in CS were coordinated and bonded with Cu^2+^ to form Cu−CS hydrogel, the peak positions of ν_N‐H+O‐H_, δ_N‐H_, and ν_C‐OH_ were shifted to different degrees. As shown in Figure 2B, the absorption peak of ν_N‐H+O‐H_ moved to 3420 cm^−1^, and the absorption peak of δ_N‐H_ disappeared, indicating that the amino group in CS was involved in the coordination of metal ions. However, the absorption peak of ν_C‐OH_ showed a split peak of 1090 and 1050 cm^−1^, respectively. Those results indicate that the hydroxyl group in the CS molecule was also involved in the coordination. When H_2_S was added to the Cu−CS hydrogel, the structure of the hydrogel was destroyed, and the peak position of FT‐IR spectra also shifted. As shown in Figure 2C, the absorption peak of ν_N‐H+O‐H_ moved to 3440 cm^−1^, and the absorption peak of δ_N‐H_ moved to 1610 cm^−1^. Those experimental phenomena show that Cu^2+^ can react with H_2_S, and Cu^2+^ competed from the hydrogel skeleton. The splitting peak of ν_C‐OH_ disappeared, that is, the absorption peak appeared at 1090 cm^−1^, which further proved that the Cu^2+^ of the chitosan hydrogel interacted with H_2_S, resulting in the dissociation of the Cu−CS hydrogel skeleton.

**Figure 3:**
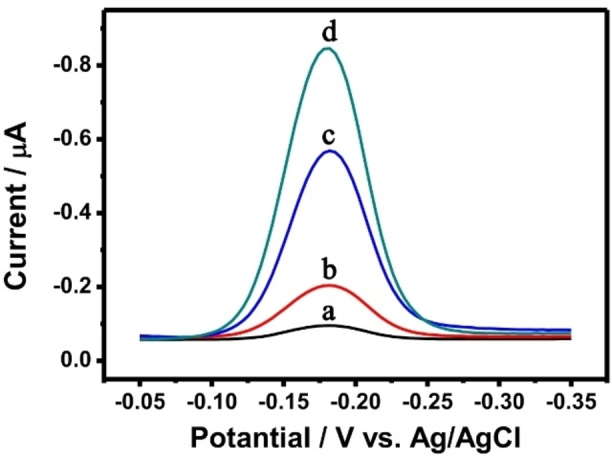
Feasibility of the Cu−CS hydrogel stimulated with different concentrations of H_2_S. a: 0 μM, b: 20 μM, c: 40 μM, and d: 60 μM.

In this experiment, MB was embedded in the hydrogel acts as an electrochemical signaling molecule. When the target was present, the hydrogel structure would be separated and MB was released. The electrochemical signal of MB was detected by the DPV method, and then the target was quantitatively analyzed and detected. As shown in Figure 3, the MB current intensity was detected using Ag/AgCl as the reference electrode and platinum wire as the counter electrode. Curve a in Figure 3 shows that the MB signal detected by Cu−CS hydrogel without H_2_S was low, and it was the same as the baseline. When 20 μM H_2_S was added, the current signal of MB increased significantly (curve b). With the increase of H_2_S concentration, the DPV current signal of the MB molecule also increased gradually. The experimental results showed that when there was no target in the Cu−CS hydrogel, MB molecules were embedded in the three‐dimensional structure of Cu−CS hydrogel, and the signal of MB molecules could not be detected in the external environment. When H_2_S was presented in the external hydrogel, Cu^2+^ in the Cu−CS hydrogel binds to H_2_S, destroying the structure of the hydrogel, and causing the MB molecules embedded in the Cu−CS hydrogel to be released. Finally, the current signal of MB was detected on the electrode, and the current signal increase with the increased of H_2_S concentration.

### Optimization of the Experimental Conditions

**Figure 4:**
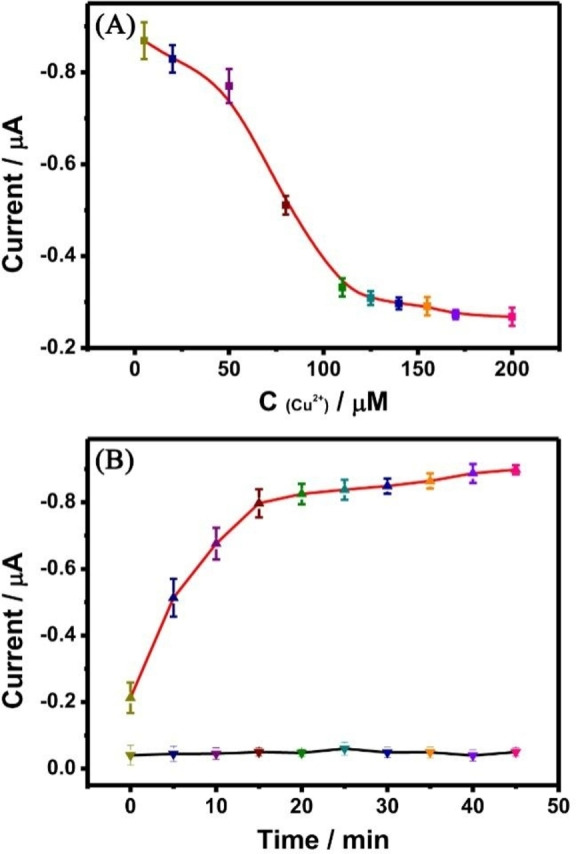
Conditions optimization for the Cu−CS hydrogel stimulated control release system: (A) the concentration of Cu^2+^; (B) The stabilization (black line) and stimulated time (red line).

In this experiment, the signal for quantitative detection of the target was derived from MB released from the hydrogel structure dissociation. Therefore, the conditions of MB release from hydrogel were optimized to achieve the best detection performance and the shortest detection time. The concentration of Cu^2+^ was optimized during the hydrogel synthesis. In this experimental, Cu^2+^ was the trigger agent for the formation of the hydrogel, the amount of Cu^2+^ in the system will significantly affect the formation of Cu−CS hydrogel; However, too much Cu^2+^ in the system will affect the detection limit of H_2_S. As shown in Figure 4A, when the system contained a low concentration of Cu^2+^, the single chain of CS polymer could not be triggered to form CS hydrogel. The free MB was detected by the DPV method. With the increased concentration of Cu^2+^, the DPV current signal of MB decreased gradually. The current signal reached a plateau at the concentration of 110 μM Cu^2+^. Therefore, the concentration of 110 μM Cu^2+^ was chosen as the optimal amount for Cu−CS hydrogel synthesis.

Subsequently, the stimuli‐responsive time of MB released and the stability of the Cu−CS hydrogel were investigated under the stimulation of 60 μM H_2_S. As shown in Figure 4B, the black curve is the stability inspection curve of Cu−CS hydrogel. According to the experimental results, the Cu−CS hydrogel synthesized in this experiment can be stably stored in the external environment within 45 min, and no obvious leakage of MB. Then, the release curve of MB molecules under the stimulation of 60 μM H_2_S was investigated (red curve). The current intensity of MB increased with the extension of time, and almost quitted increasing after 15 minutes, indicating that the structure of Cu−CS hydrogel was separated at this time. Therefore, 15 min was selected as the optimum release time for subsequent experiments.

### Properties of Cu−CS Hydrogel

**Figure 5:**
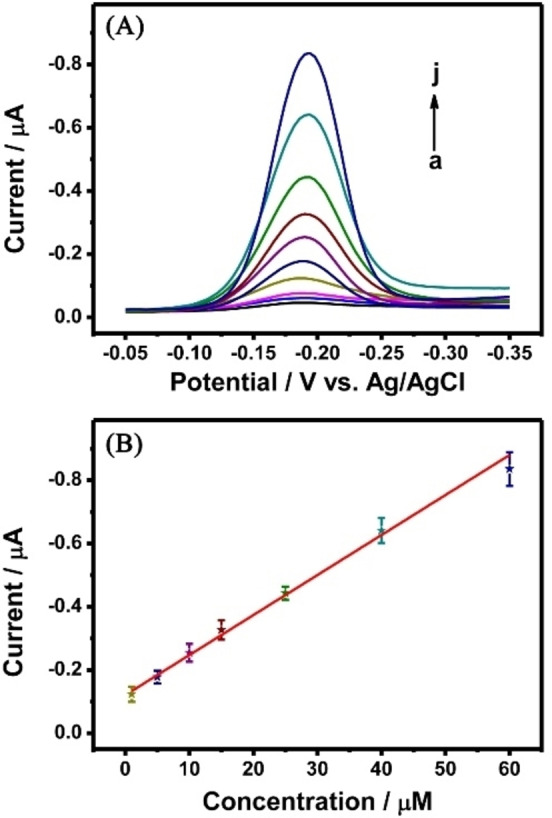
(A) The DPV responses at different concentrations of H_2_S were stimulated with Cu−CS hydrogel. a~j: 1, 5, 10, 15, 25, 40, 60 μM H_2_S; (B) The relationship between the DPV current and the Na_2_S concentration.

Based on the above optimized experimental conditions, different concentrations of H_2_S were selected to investigate the performance of Cu−CS hydrogel for quantitative detection. As shown in Figure 5A, the concentration of MB released from the hydrogel gradually increased with increasing concentration of the target, and the current signal detected through the DPV gradually increased. A linear fit between the different concentrations of the target from 1 to 60 μM and the current intensity revealed a good linear relationship (Figure 5B). The linear equation is as follows.

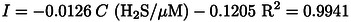




Where *I* is the current intensity of MB, *C* is the concentration of target H_2_S, and R^2^ is the linear correlation coefficient. It satisfies the current common Na_2_S concentration analysis and detection in biological samples.

**Figure 6:**
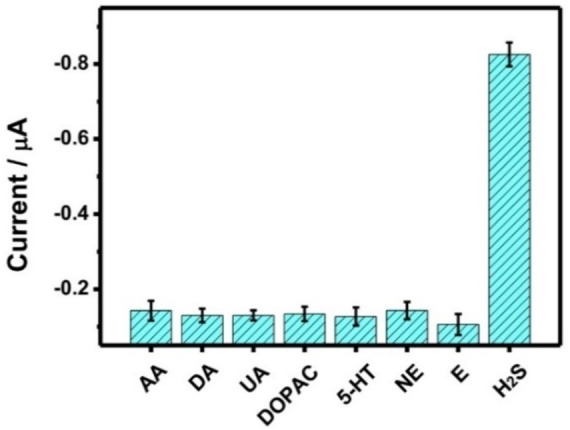
Selectivity of the proposed Cu−CS hydrogel for Na_2_S detection.

To investigate the specific response performance of the prepared Cu−CS hydrogel to H_2_S, some common physiological active substances were selected to investigate its stimulus‐response performance. As shown in Figure 6, the selected concentrations of interfering substances were 400 μM ascorbic acid (AA), 20 μM dopamine (DA), 20 μM uric acid (UA), 50 μM dihydroxy‐phenyl aceticacid (DOPAC), 50 μM 5‐hydroxytryptamine (5‐HT), 10 μM norepinephrine (NE), and 10 μM epinephrine (E). The experimental results show that the DPV current signal measured by common interfering substances was similar to the blank value signal. Only in the presence of the target, the Cu−CS hydrogel could produce a stimulus‐response and release MB. Therefore, the Cu−CS hydrogel prepared in this method showed good stimulus response performance to H_2_S.

### Application of the Proposed Cu−CS Hydrogel

Table 1 lists the results obtained using the proposed method. Compared with the normal human serum samples, the samples from the PIH patients resulted in a significant statistical difference. The standard addition recovery rate was 91.83–95.62 % in this range by proposed Cu−CS hydrogel with acceptable relative standard deviation values. It can be concluded that the Cu−CS hydrogel is efficiently used for the detection of H_2_S in biological samples.

**Table 1:** Determination of H2S in human serum samples using the proposed Cu−CS hydrogel (n=3).

Sample	Detected (μM)		Spiked (μM)	Total Found (μM)	Recovery (%)
Normal Human serum	1	44.58±3.31	10.00	52.02±3.57	95.31
	2	41.95±3.17	10.00	39.55±3.20	94.28
	3	42.34±3.52	10.00	50.04±3.35	95.62
PIH Patients serum	1	21.62±3.91	10.00	29.17±3.42	92.26
	2	24.22±2.74	10.00	31.42±3.83	91.83
	3	19.82±3.27	10.00	27.27±3.66	91.45

## Conclusions

In this paper, the Cu−CS hydrogel embedded with MB was prepared by introduced metal ions (Cu^2+^) as the cross‐linking agent. The Cu^2+^ coordinated with the amino and hydroxyl groups in CS, resulting in the polymerization of the single chain of CS polymer and the formation of Cu−CS hydrogel. The synthesis process of Cu−CS hydrogel takes only 5 s, which has the advantages of rapid and simple synthesis. Due to Cu^2+^ binding to H_2_S, the K_sp_ constant of the generated CuS is extremely low. Therefore, when H_2_S is contained in the external environment, it can generate a stimulus‐response with Cu−CS hydrogel, leading to the destruction of the hydrogel structure and the release of MB. By detecting the electrochemical signal of MB, the quantitative detection of H_2_S can be realized.

## Conflict of Interests

The authors declare no conflict of interest.

1

## Data Availability

Research data are not shared.
